# The myokine musclin in metabolic syndrome: Pathological links and exercise interventions

**DOI:** 10.1016/j.isci.2025.114367

**Published:** 2025-12-16

**Authors:** Ruiming Wen, Yuan Yang, Haixia Wang, Yikun Teng, Bing Zhao, Hanxiao Zhu, Songtao Wang

**Affiliations:** 1 School of Physical Education and Sports Science, South China Normal University, Guangzhou 510006, China; 2 Laboratory of Regenerative Medicine in Sports Science, School of Sports Science, South China Normal University, Guangzhou 510006, China; 3 School of Physical Education, Shenzhen University, Shenzhen 518060, China

**Keywords:** Therapeutics, Disease, Mechanism of action, Interventions, Physiology

## Abstract

Since its identification in 2004, the myokine Musclin, a skeletal muscle-specific secretory factor, has garnered increasing attention in the fields of metabolism and exercise medicine due to its pleiotropic regulatory functions. This review proposes and substantiates the central thesis that Musclin acts as a “bidirectional hub” connecting exercise and metabolic homeostasis. Under physiological conditions, the pulsatile secretion of Musclin promotes mitochondrial biogenesis and enhances exercise endurance. In contrast, during pathological states, its overexpression exacerbates metabolic disorders by interfering with insulin signaling, inducing endoplasmic reticulum stress (ERS), and suppressing adipose thermogenesis. A body of evidence indicates that the expression and function of Musclin are precisely regulated by genetic, nutritional, and exercise-related factors, underscoring its pivotal role in the systemic metabolic network. Although its elevated levels may be perceived as a compensatory response in certain contexts, gain-of-function experiments and other evidence posit that Musclin primarily acts as a “pathological driver,” demonstrating context-dependent effects in obesity, type 2 diabetes mellitus (T2DM), hypertension, and other components of metabolic syndrome (MetS). Current research in the field faces challenges, including sample heterogeneity, lack of standardized detection methods, and a translational gap between animal models and human diseases. Therefore, this review systematically integrates the molecular characteristics, pathophysiological effects, and exercise adaptation mechanism of Musclin, and reveals its “bidirectional hub” role in metabolic homeostasis.

## Introduction

Recent studies show that skeletal muscle is not only the core executive organ of the locomotor system, but also an important endocrine tissue. It mediates inter-organ communication by secreting myokines and serves as an upstream regulatory hub for the local and systemic adaptive responses induced by exercise.^1^^,^^2^ Among these, the “exerkines” hypothesis proposes that myokines released by skeletal muscle during contraction activities (such as exercise, and so forth) act on local tissues in an autocrine/paracrine way, or remotely regulate metabolic organs such as liver, adipose tissue, heart, brain and vascular system in an endocrine way, and construct an inter-organizational metabolic regulation network, thus affecting the occurrence of systemic metabolic diseases.^3^^,^^4^^,^^5^

In 2004, Nishizawa et al. identified Musclin from mouse skeletal muscle for the first time and expressed it in skeletal muscle with high specificity.^6^ Unlike canonical myokines such as Irisin and interleukin (IL)-6, Musclin possesses a unique domain exhibiting high homology to the natriuretic peptide (NP) family, as well as a putative KKKR serine protease cleavage site, a hallmark feature of NP proteins. This structural configuration suggests its potential role in coordinating energy metabolism and cardiovascular function.^6^^,^^7^ Given the close relationship between skeletal muscle and whole-body metabolism, this review focuses on elucidating the function of Musclin within the complex pathological context of metabolic syndrome (MetS). MetS is defined as a cluster of metabolic disorders, characterized by aberrations in the metabolism of proteins, fats, and carbohydrates. Its core components include abdominal obesity, insulin resistance (IR), dyslipidemia, and elevated blood pressure, which collectively significantly increase the risk of developing type 2 diabetes mellitus (T2DM) and cardiovascular disease.^8^ Specifically, Musclin affects the whole-body energy homeostasis by regulating adipose tissue thermogenesis, energy metabolism, lipid deposition, interfering with insulin signal transduction, and inducing endoplasmic reticulum stress (ERS), and may become a new target for the treatment of MetS.^9^^,^^10^ However, its role in hypertension presents a paradox, exhibiting species and disease-stage dependent contradictions.^11^^,^^12^ These discrepancies may stem from dynamic “compensation-decompensation” shifts during different disease stages or inherent differences in cardiovascular regulatory systems across species. Furthermore, as a key mediator of exercise adaptation, the dynamic secretion pattern of Musclin is regulated by genetic polymorphisms, nutritional status, and exercise modality.^13^^,^^14^^,^^15^^,^^16^^,^^17^ It contributes to the maintenance of muscle mass by inhibiting fibroadipose progenitor cells (FAP) proliferation^18^ and enhances cardiac mitochondrial function to confer protection against ischemic injury.^19^

Consequently, critical aspects such as the threshold effect governing its pathophysiological switch, the mechanisms of its inter-organ interactions, and its clinical translational potential require more systematic investigation. This review integrates recent research advances to systematically elaborate on the molecular characteristics of Musclin, its dual roles in MetS, and its pivotal function in exercise adaptation, thereby offering new perspectives for the precise prevention and treatment of MetS.

## Overview of musclin

### Distribution and structure of musclin

In 2004, the Japanese scholar Nishizawa H. and his team first identified this protein from mouse skeletal muscle and, owing to its muscle-specific expression pattern, named it Musclin.^6^ Its expression level in skeletal muscle is more than 10-fold higher than in bone, brown adipose tissue, testis, and spleen.^20^ Musclin transcription is most abundant in type IIb muscle fibers, followed by type IIx, and is relatively low in type IIa and I fibers. This expression profile, which is strongly associated with a fast-glycolytic phenotype,^21^ suggests that its function may be closely linked to anaerobic metabolism and rapid energy mobilization. Subsequent research revealed that Musclin shares the same gene coding sequence as osteocrin. Through the use of alternative promoters and differential splicing, two isoforms are generated: osteocrin, which is highly expressed in bone tissue during the embryonic stage, and Musclin, which is specifically expressed in adult skeletal muscle. This mechanism reveals a functional switch of the gene during development.^15^^,^^22^^,^^23^^,^^24^^,^^25^

**Figure 1:**
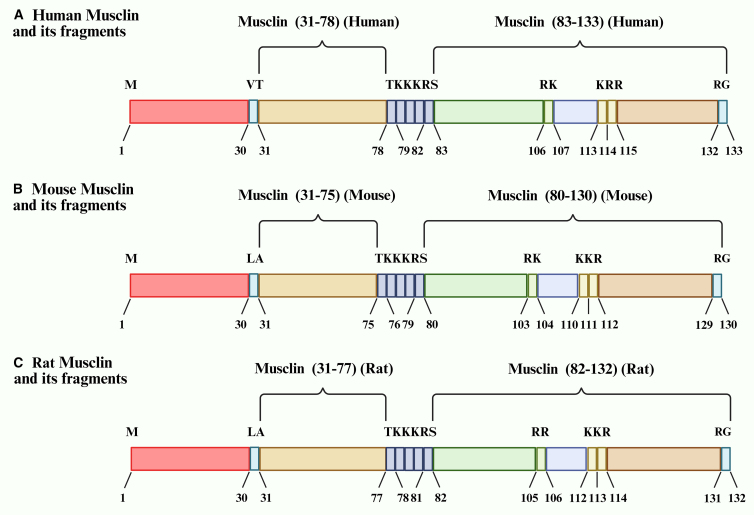
Musclin and its fragments in various species (A–C) Musclin and its fragments in humans; (B). Musclin and its fragments in rats; (C). Musclin and its fragments in the mouse.

Mouse-derived Musclin protein is composed of 130 amino acids (molecular weight is about 11 KDa), and its N-terminal 27 amino acids are signal peptides, which are secreted to form a mature protein (MUS-F) with 103 amino acids.^6^^,^^20^ The protein contains two KKKR serine protease cleavage sites and a region with high homology to the NP family,^26^ while Musclin does not have two cysteine residues required to form NPΩ-like structural characteristics.^20^ C2C12 myoblast experiment confirmed that Musclin can be cut into full-length and truncated forms after secretion, suggesting that the KKKR site is involved in post-translational processing.^6^ The protein belongs to a hydrophilic, unstable mixed structure, and the secondary structure includes α-helix (35%), β-turn (18%), and random coil (47%). Although the overall amino acid homology between mouse, human, and rat is 75.2% and 90.2%, respectively,^27^ the downstream region of the KKKR cleavage site at positions 81–130 (containing NP homology domain) is highly conserved, and there is only 1–2 amino acid difference across species. This core sequence is named mus33,^28^ which is speculated to be the key functional domain mediating NP-related signaling. It may participate in physiological processes such as energy metabolism and muscle-bone interaction through competitive receptor binding (Figure 1). The conservation of this domain not only provides a basis for cross-species functional studies but also implies its indispensable role in the core life process of energy homeostasis, thereby establishing a structural biology foundation for its involvement in MetS.

### The distinct niche of musclin within the myokine network

Skeletal muscle-derived myokines work in concert through a complex network to co-regulate metabolic homeostasis and exercise adaptation.^29^^,^^30^ Although numerous myokines participate in modulating metabolism and mitochondrial function, Musclin exhibits distinct uniqueness in its molecular structure, signaling pathways, and functional outputs.

First, regarding molecular structure and signaling, Musclin is unique due to its characteristic NP-homology domain. This distinct structure suggests a signaling mechanism divergent from that of Fractalkine/CX3C chemokine ligand 1 (CX3CL1). CX3CL1 primarily exerts its effects through the specific receptor CX3CR1, functioning as a chemokine that is more focused on regulating intramuscular inflammatory responses, angiogenesis, and mitochondrial dynamics/quality control in response to energetic stress.^31^^,^^32^ In contrast, secreted protein acidic and rich in Cysteine (SPARC) mediates cell-matrix interactions mainly via integrins and related cell surface receptors, influencing insulin sensitivity and glucose metabolism. Consequently, SPARC’s impact on mitochondria is achieved more indirectly through the improvement of the systemic metabolic environment.^33^

Secondly, regarding core metabolic targets and regulatory mechanisms, the function of Musclin presents an intriguing contrast to that of C1q tumor necrosis factor-related protein 15 (CTRP15). Both are involved in lipid metabolism regulation: CTRP15 has been shown to promote fatty acid uptake and lipid synthesis in adipocytes, primarily functioning in lipid storage and allocation.^34^ In contrast, recent studies on Musclin demonstrate that it binds to the transferrin receptor 1 (Tfr1) receptor on beige adipose tissue, directly suppresses the cAMP/PKA signaling pathway and thermogenesis, thereby acting as a “brake” in energy balance—a role distinct from that of CTRP15.^9^ This direct inhibition of adipose thermogenesis represents a key characteristic that distinguishes Musclin from other myokines known to promote thermogenesis or lipid mobilization, such as Irisin.

Finally, in exercise-induced adaptive responses, these myokines play distinct roles. CX3CL1 and SPARC are primarily associated with tissue repair, inflammation balance, and extracellular matrix remodeling. Reported changes in CTRP15 levels following exercise remain inconsistent.^35^^,^^36^ In comparison, Musclin is well-established as an “exercise-responsive factor,” whose acute elevation is essential for activating the cGMP/PGC-1α axis, promoting mitochondrial biogenesis, and enhancing endurance performance.^15^

In summary, compared to other myokines such as CX3CL1, SPARC, and CTRP15, Musclin occupies a non-redundant niche within the myokine network, attributable to its unique NP homology domain, its direct inhibitory effect on adipose thermogenesis, and its central role in exercise-induced mitochondrial biogenesis (Table 1). This comparative analysis not only underscores the uniqueness of Musclin but also reinforces its potential as a therapeutic target for MetS.

**Table 1:** Comparison of musclin with other metabolism related myokines

Feature	Structural Motif	Primary Expression	Core Pathway	Effect on Adipose Tissue	Mitochondrial Effect
Musclin	NP-like domain	Skeletal muscle (predominantly fast-twitch fibers)	Tfr1, NPR-C, cGMP/PKA	Inhibits beige adipose thermogenesis	Promotes biogenesis (via PGC-1α)
CX3CL1	Chemokine	Skeletal muscle, endothelial cells	CX3CR1	Modulates adipose tissue inflammation	Regulates dynamics and quality control
SPARC	Acidic/CR-rich	Skeletal muscle, osteoblasts	Integrins, CD36	Influences adipocyte differentiation	Indirect improvement (via systemic metabolism)
CTRP15	C1q/TNF-like	Skeletal muscle, adipose tissue	Unknown receptor	Promotes fatty acid uptake and lipid storage	Not well characterized

## Musclin and obesity

Obesity, particularly central obesity, constitutes the pathological initiator and a core diagnostic criterion of MetS. It is defined as a state of excessive fat accumulation that impairs health,^37^^,^^38^ a condition typically resulting from a chronic imbalance between energy intake and expenditure or from alterations in body metabolism that lead to excessive weight gain.^39^^,^^40^ Musclin, as a muscle-specific secretion factor, plays a complex and key role in obesity and related metabolic disorders. The existing research reveals the duality of its function: on the one hand, its expression level is positively correlated with the degree of obesity, suggesting that it may be used as a biomarker or pathogenic factor of obesity; On the other hand, exogenous intervention experiments show that it has the potential to inhibit obesity. Its specific effect may depend on the body’s metabolic state, target, and regulatory background.^9^^,^^28^^,^^41^^,^^42^^,^^43^^,^^44^

Clinically, the content of Musclin in plasma and skeletal muscle is significantly elevated in obese individuals, showing positive correlations with key indicators such as body mass index (BMI), triglycerides (TC), fasting Plasma Glucose (FPG), and IR.^9^^,^^41^^,^^42^ This correlation is consistently recapitulated in animal models, including high-fat diet-induced obese mice, genetically obese mice, and obese SD rats, all exhibiting the upregulation of Musclin expression in skeletal muscle.^9^^,^^41^ These observational evidences show that there is a stable correlation between the expression of Musclin and obesity, which is strong evidence that musclin is a biomarker of disease. Secondly, functional experiments revealed the molecular mechanism of Musclin actively promoting obesity by inhibiting adipose heat production and energy consumption. Mechanism research shows that over-expression of Musclin can significantly inhibit the thermogenic function of beige adipose in mice, which is manifested in lipid decomposition, glucose metabolism, and down-regulation of key thermogenic genes, which eventually leads to the reduction of whole body energy consumption and aggravation of obesity and related metabolic abnormalities.^9^ The core mechanism is that Musclin directly binds to Tfr1 on beige adipocyte membrane, antagonizing the signal pathway of cAMP/PKA, thus inhibiting thermogenesis.^9^ In addition, Musclin can also regulate the metabolism and fate of adipocytes, on the one hand, it can weaken lipid accumulation, on the other hand, it can enhance lipolysis and apoptosis, which is mediated by PKA/p38 MAPK pathway,^42^ and may involve the regulation of peroxisome proliferator-activated receptor γ(PPARγ) and liver X receptor α(LXRα) pathways.^44^

Finally, the expression of Musclin is precisely regulated by epigenetics and transcription factors, and exogenous intervention shows its potential as a therapeutic target. In the regulatory mechanism, G9a methylase inhibits the expression of Musclin through histone modification transcription, while phosphorylation of transcription factor FOXO1 can up-regulate its expression.^28^ The most enlightening thing is that, contrary to the pathogenic effect of endogenous Musclin, exogenous injection of recombinant Musclin or its functional core peptide can effectively inhibit obesity and hepatic steatosis induced by high-adipose diet.^28^ This seemingly paradoxical phenomenon underscores the complexity of Musclin’s biological functions, indicating that its net effect may be highly context-dependent and spatiotemporally specific. The discrepancy may stem from differential signaling outcomes: the chronic, low-grade elevation of endogenous Musclin during obesity progression may lead to receptor desensitization or compensatory adaptations, allowing its thermogenesis-inhibiting effect to dominate. In contrast, acute exogenous supplementation might represent a potent intervention that preferentially activates alternative beneficial pathways (e.g., stimulating lipolysis), thereby overriding its thermogenesis-suppressive effects in the short term. The divergent consequences of chronic versus acute exposure not only highlight the functional complexity of Musclin but also suggest that within the obesity component of MetS, it may transition from an early compensatory signal to a late-stage pathogenic factor.

**Figure 2:**
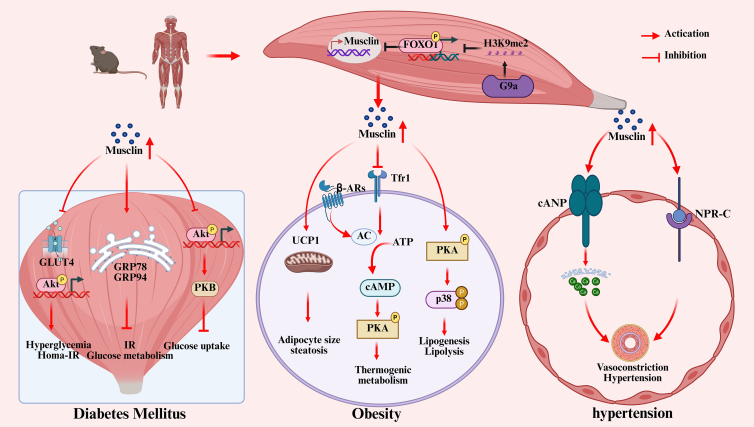
Molecular mechanism diagram of musclin in various MetS

In a word, Musclin, as a muscle-specific secretory protein, plays an important role in the pathogenesis of obesity. Musclin affects the whole-body energy homeostasis by regulating heat production, energy metabolism, and lipid deposition of adipose tissue, and may become a new target for obesity treatment (Figure 2). Future research will further reveal the biological function of Musclin and its potential application in the treatment of obesity and related metabolic diseases.

## Musclin and type 2 diabetes mellitus

T2DM represents a major clinical outcome of MetS, characterized by hyperglycemia resulting from the combined effects of IR and pancreatic β-cell dysfunction.^45^^,^^46^ Chronic hyperglycemia in T2DM is associated with long-term damage, dysfunction, and eventual failure of various organs, particularly the kidneys, heart, and blood vessels.^47^^,^^48^ It has been reported that Musclin can inhibit the glucose uptake process of myoblasts induced by insulin,^6^ which indicates that Musclin, a muscle secretion factor, has complex regulatory characteristics in the occurrence and development of T2DM and complications: on the one hand, it is a “pathological factor” directly involved in the occurrence of diseases, on the other hand, it may be a “compensatory marker” reflecting metabolic disorders.

A lot of evidence shows that Musclin, as a “pathological factor,” directly promotes IR by damaging the insulin signaling pathway. Clinical studies have found significantly elevated Musclin levels in patients with diabetic nephropathy (DN), which increase with disease progression.^49^ Moreover, Musclin levels are markedly higher in newly diagnosed, patients with treatment-naïve T2DM, showing positive correlations with fasting glucose, insulin, TC, and the homeostasis model assessment of IR (HOMA-IR) index.^50^ Consistently, in T2DM rats, Musclin expression negatively correlates with skeletal muscle Akt phosphorylation and GLUT4 translocation levels,^51^^,^^52^ indicating its involvement in T2DM pathogenesis likely through modulating key molecules in the insulin signaling pathway. In addition, recombinant Musclin significantly weakened glucose uptake and glycogen synthesis in muscle cells stimulated by insulin.^6^ When Musclin is directly incubated with normal rat skeletal muscle, it can inhibit the uptake of 2- deoxyglucose (2-DG) and the expression of GLUT4, at the same time, it can up-regulate the ERS marker GRP78/GRP94 and reduce the phosphorylation of Akt/PKB, forming a vicious cycle of “Musclin- ERS-impaired insulin signal.”^41^^,^^44^ It is worth noting that glucosamine (GlcN) activates the key pathways of unfolded protein reaction (UPR) by inducing ERS, and significantly upregulates the expression of *Musclin* gene in skeletal muscle, thus interfering with the insulin signaling pathway to induce IR, and finally leading to persistent hyperglycemia in mice. This mechanism has been proven to be an important pathological link of IR induced by GlcN in mice.^10^

On the other hand, changes in Musclin levels may also reflect an adaptive response to metabolic disturbances, suggesting its role as a “compensatory marker.” One study reported that Musclin levels in patients with DN are sex-dependent (higher in females than males) and increase significantly with DN progression.^49^ Supporting this, ovariectomized mice exhibit markedly increased Musclin expression in skeletal muscle,^53^ potentially mediated by estrogen receptors acting directly on the gene promoter or through membrane receptor-activated signaling pathways to enhance its transcription and secretion. This regulatory mechanism may explain the observed higher Musclin levels in both female patients with DN and elite female athletes.^50^ Additionally, a recent study in middle-aged and elderly patients with T2DM found no direct correlation between blood glucose and serum Musclin levels, which might be attributed to the masking effect of anti-diabetic medications on their direct relationship,^54^ possibly indicating compensatory adaptation under different physiological or pharmacological conditions. Overall, within the T2DM component of MetS, Musclin primarily demonstrates its “pathology-driving” nature, yet its potential as a compensatory stress signal should not be overlooked.

To sum up, Musclin mainly plays the role of “pathological factor” in DM, which promotes the disease progression by directly interfering with insulin signal and inducing ERS; At the same time, the change of its expression level may also reflect the complex compensatory response of the body to metabolic disorder (Figure 2). Future research needs to further analyze the regulation mechanism of gender differences, the influence of drug intervention, and the specific role in diabetic complications, to provide a new direction for developing DM treatment strategies targeting Musclin.

## Musclin and hypertension

Hypertension is a core diagnostic component of MetS and, together with IR and obesity, forms a pathophysiological network. Defined as a chronic cardiovascular syndrome characterized by persistently elevated systemic arterial pressure,^55^^,^^56^ hypertension is typically diagnosed when systolic blood pressure ≥140 mmHg and/or diastolic blood pressure ≥90 mmHg, and is classified into primary and secondary types.^57^ As a leading global modifiable burden of chronic disease, its pathological progression involves multifaceted mechanisms, including vascular remodeling, activation of the renin-angiotensin system, sympathetic overactivity, and disturbances in the metabolic-inflammation axis.^58^^,^^59^^,^^60^ Therefore, examining the role of Musclin in hypertension from the holistic perspective of MetS holds significant pathophysiological relevance.

Recent studies indicate that skeletal muscle loss is recognized as an independent risk factor for the development of hypertension,^61^ and myokines secreted by skeletal muscle may play different roles in hypertension through the mutual adjustment of the adipose-muscle axis.^11^ Clinical studies have found that there is a complex correlation between the circulating level of Musclin and the course of hypertension: the circulating level of Musclin in patients undergoing transcatheter aortic valve implantation is low, often accompanied by weakness, low albumin, hypertension and stroke history^62^; The plasma Musclin level in patients with essential hypertension is generally lower than that in healthy people, but it is significantly higher in patients with hypertension-related stroke.^11^ This biphasic feature of “low basal level-pathological compensatory increase” suggests that Musclin may play a different role in different stages of hypertension (such as vascular homeostasis vs. end organ injury), or be regulated by secondary factors such as inflammation and oxidative stress.

In animal experiments, on the contrary, the expression of Musclin in the aortic tissue of spontaneously hypertensive rats and the arterial tissue of spontaneously hypertensive rats induced by DOCA-Salt was significantly higher than that of the normal group,^12^^,^^63^ and the vasoconstriction in the aortic strips isolated from normal rats or spontaneously hypertensive rats was induced in a concentration-dependent manner, especially in spontaneously hypertensive rats,^12^ which seemed to be the direct effect of Musclin on intracellular calcium increase.^63^ Exogenous tail vein injection of Musclin can directly participate in the regulation of vascular tone by binding to receptors such as NPR-C, thereby increasing systolic blood pressure.^12^

Although the above evidence confirms an association between Musclin and hypertension, a notable “clinical-animal model paradox” persists, underscoring the necessity for critical appraisal. As summarized in Table 2, clinical studies generally report reduced baseline circulating Musclin levels in patients with essential hypertension,^11^^,^^62^ whereas various hypertensive animal models consistently show the upregulation of Musclin in vascular tissues and demonstrate its direct vasoconstrictive function.^12^^,^^63^ This apparent contradiction may arise from several confounding factors. First, inherent differences may exist between humans and rodents in the neurohumoral regulation of the cardiovascular system, as well as in the distribution of Musclin receptors and their downstream signaling pathways. Second, multiple circulating isoforms of Musclin may possess different biological activities. Variations in the recognition efficiency of detection methods for these different isoforms could directly affect the interpretation of results. Most importantly, clinical studies often involve patients with long-standing, diagnosed hypertension who frequently present with multiple comorbid metabolic disorders. Their low Musclin levels may reflect a state of “compensatory exhaustion” in the vascular system under persistent metabolic stress. Conversely, animal models typically simulate early-stage or acute phases of the disease, during which the upregulation of Musclin might represent an initial compensatory mechanism to maintain perfusion pressure. The compensatory elevation of Musclin observed in patients with hypertension-related stroke further supports the hypothesis that its function dynamically shifts with disease progression.^11^

**Table 2:** Expression and function of musclin in clinical studies and animal models of MetS

Pathological model	Musclin expression level	Pathological Phenotypes	References
HFD mice、obese people	up-regulation	BMI was positively correlated.	Jin et al.^9^
SD rats, overweight/obese people	up-regulation	The levels of DBP, TG, fasting serum insulin, and HOMA-IR were higher.	Chen et al.^41^
MetS population	up-regulation	It is not related to BMI, but positively related to lean body mass, insulin, blood sugar, visceral fat, and muscle mass.	Sánchez et al.^43^
Newly diagnosed untreated patients with T2DM	up-regulation	FPG, insulin, TC, and HOMA-IR increased.	Chen et al.^49^
Patients with DN	up-regulation	It was positively correlated with body mass index, systolic blood pressure, blood urea nitrogen, creatinine, and ACR.	Zhang et al.^50^
Middle-aged and elderly patients with T2DM	no change	BMI, gait speed, grip strength, skeletal muscle mass, and ASM are positively correlated.	Fuet al.^54^
T2DM rats	up-regulation	FPG and insulin levels increased.	Shimomura、Yu et al.^51^^,^^52^
Patients undergoing transcatheter aortic valve implantation	down-regulation	The prevalence of weakness, low albumin, hypertension, and stroke increased.	Kattih et al.^62^
Patients with essential hypertension	down-regulation	BMI, SBP, FBG, and HbA1c increased, while HDL-C and eGFR levels, exercise frequency and duration decreased.	Chen et al.^11^
Hypertension-related stroke patients	up-regulation	The exercise frequency decreases.	Chen et al.^11^
Aorta of spontaneously hypertensive rats	up-regulation	Vasoconstriction	Li et al.^12^
Arteries in hypertensive rats induced by DOCA-Salt	up-regulation	elevation of blood pressure	Lin et al.^63^

Therefore, Musclin should not be simplistically categorized as a “pro-hypertensive” or “anti-hypertensive” factor; its net effect is profoundly context-dependent. This paradox highlights the complexity of Musclin as a key mediator in the metabolic-vascular axis: acute elevation under physiological or early pathological conditions may contribute to homeostasis by modulating vascular tone, whereas chronic dysregulation of its signaling in the setting of MetS may ultimately lead to vascular dysfunction. Future research should focus on elucidating the dynamic changes of Musclin across different stages of hypertension, its concentration-dependent effects, and its interactions with vascular remodeling and the inflammatory microenvironment, in order to clarify its potential value as a therapeutic target.

## Musclin in exercise adaptation and its dynamic secretion patterns

### Musclin as a key regulator of exercise adaptation

Skeletal muscle produces and secretes myokines, which mediate local and systemic cross-talk to promote exercise tolerance and overall health.^64^^,^^65^^,^^66^ In the forced swim test, intraperitoneal injection of Musclin reduced immobility time and increased swimming distance in mice.^67^ Substantial research has identified Musclin as an exercise-responsive factor that promotes mitochondrial biogenesis in skeletal muscle and enhances exercise endurance, thereby improving physical performance in mice.^15^ Mice lacking Musclin exhibit reduced physical capacity and display diminished activity-dependent, atrial NP(ANP)/cGMP/PGC-1α dependent mitochondrial biogenesis in skeletal muscle,^15^ though lipid mobilization remains unaffected.^16^

However, a key unexplored area is whether exercise-induced Musclin further influences mitochondrial dynamics and mitophagy. It is hypothesized that Musclin may optimize mitochondrial network morphology by regulating the activity of dynamin-related protein 1 (DRP1) and optic atrophy 1 (OPA1), thereby meeting energy demands more efficiently during exercise. Concurrently, it may facilitate the clearance of damaged mitochondria via the PTEN-induced putative kinase 1 (PINK1)/Parkin pathway, helping to maintain metabolic quality control in skeletal muscle. Given that type II (fast-twitch) skeletal muscle fibers inherently possess lower mitochondrial density and oxidative capacity than type I (slow-twitch) skeletal muscle fibers,^21^ the high expression of Musclin in type II skeletal muscle fibers may represent a compensatory or adaptive mechanism. By strongly driving mitochondrial biogenesis and functional optimization, Musclin could help offset the inherent aerobic metabolic limitations of these fibers, thereby supporting their performance during high-intensity exercise. Furthermore, current understanding of the specific effects of different exercise modalities (such as endurance training versus resistance training) on Musclin expression remains limited. There is a clear need for in-depth analysis using muscle-specific knockout models in combination with tailored exercise regimens. This line of inquiry opens new research directions for understanding how exercise improves cellular energy metabolism via myokines.

### Dynamic secretion pattern of musclin

The levels of Musclin are influenced by various factors, including exercise, diet, and environmental temperature. Produced and secreted by skeletal muscle into the systemic circulation, Musclin exhibits a dynamic secretory pattern characterized by a rapid increase immediately after acute exercise, peaking around 15 min post-exercise, followed by a decline during recovery (measured at 60 and 120 min). This pattern is consistent with that of a typical “exercise factor.”^16^^,^^17^

Under long-term exercise adaptation, both skeletal muscle and circulating Musclin levels persistently deviate from the normal range. In experienced marathon runners, Musclin levels of serum decrease immediately after the race and remain reduced at 24 and 72 h post-race,^14^^,^^68^ accompanied by elevated markers of muscle damage. Individuals carrying the BDKRB2 −9 or ACE D alleles exhibit a more pronounced post-race reduction in Musclin, along with a stronger cytokine response associated with muscle repair and cardioprotection.^13^ Such persistent pathological stimulation may lead to the desensitization or remodeling of downstream signaling pathways. For instance, regular exercise significantly reduces the proliferation of FAPs and upregulates Musclin expression, thereby attenuating collagen deposition and fat infiltration in injured or disuse-induced muscle atrophy. Mechanistically, Musclin suppresses FAP proliferation and promotes their apoptosis by upregulating FILIP1L—a process partly dependent on the transcription factor FoxO3a.^18^

In addition, Musclin is positively correlated with carbohydrate intake.^14^ Runners with adequate carbohydrate intake have higher levels of Musclin, which is helpful to improve their exercise adaptability.^14^ Kysel et al. found that compared with a periodic ketogenic diet (CKD), balanced diet (RD) combined with regular resistance/aerobic training significantly increased Musclin level.^69^ *In vitro* experiments showed that during the differentiation of C2C12 myotubes, the mRNA expression of Musclin increased significantly in a time-dependent manner, and was easily regulated by environmental temperature. Thermal neutrality significantly increased the expression of Musclin at 30°C.^9^ The above report means that the expression level of Musclin presents a dynamic secretion pattern, which is closely related to exercise and nutritional level (Table 3).

**Table 3:** Characteristics of the dynamic secretion pattern of musclin

Intervention measures	Objects	Intervention conditions	Musclin	References
Exercise	C57BL/6 mice	12 m/min、tilt 15°、5 days/w、45 min/d,1w	up-regulation	Subbotina et al.^15^
Exercise	21 inactive young men	70-75%HRmax, Once	up-regulation during exercise, down-regulation during recovery	Nam et al.^16^
Exercise	110 men	24w training plan: endurance exercise: high intensity, 80% HRres, 150min; medium strength, 60%HRres, 150min. Strength exercise: moderate intensity, 50% 1-RM, high intensity 70% 1RM, 80min.	up-regulation	Mendez et al.^17^
Exercise	C57BL/6 mice	11 m/min, tilt 15°, 5days/w, 60 min/d, 6w	up-regulation	Kang et al.^18^
Exercise	57 Brazilian amateur male marathon runners	≥30km/w	down-regulation	Sousa et al.^68^
Diet	74 Brazilian amateur male marathon runners	Carbohydrate intake (g/kg) (>5 g/kg/day)	up-regulation	Sierra et al.^14^
Diet	25 healthy young men	Balanced nutrition and reduced diet: 55% carbohydrates, 30% fat, and 15% protein	up-regulation	Kysel et al.^69^
Gene	74 Brazilian amateur male marathon runners	Carrying BDKRB2-9 allele or ACE D allele	down-regulation	Sierra et al.^13^
Ambient temperature	C57BL/6 mice	Hot neutral at 30° C, 1w	up-regulation	Jin et al.^9^

## Musclin as a key mediator of exercise-induced amelioration of metabolic disorders

Under MetS conditions, Musclin exhibits aberrantly high expression. This phenomenon may not merely be a pathological marker but rather a compensatory adaptive response of the organism to metabolic stress.^9^^,^^41^ Based on current evidence, we propose that Musclin may function as a “metabolic stress factor”: during the early stages of metabolic disturbance, its upregulated expression represents an adaptive compensatory mechanism initiated by the body to restore homeostasis, aiming to exert positive metabolic regulatory effects.^28^^,^^42^ However, when pathogenic factors (such as a long-term high-calorie diet) persist, the Musclin signaling pathway may be “resistant”-that is, although its circulation level continues to increase, its downstream beneficial physiological effects gradually weaken or fail,^9^ which is similar to the mechanism of IR.^44^

Long-term training fundamentally improves metabolic status, reduces the body’s “demand” for compensatory high Musclin expression, and ultimately normalizes its levels.^17^ Specifically, long-term aerobic exercise reduces Musclin expression in the skeletal muscle of obese rats while improving lipid metabolism and insulin sensitivity.^52^ Similarly, an 8-week resistance exercise program significantly lowered Musclin levels in the skeletal muscle of T2DM rats and improved metabolic parameters by activating the Akt/GLUT-4 signaling pathway.^51^ High-intensity interval training has been shown to reduce serum Musclin levels more effectively than continuous aerobic exercise in patients with MetS, and this reduction correlates with improved IR.^70^ Notably, Musclin itself possesses important health-promoting effects, with its functionality depending more on its dynamic expression pattern than on its absolute levels. Studies have found that HIIT reduces Musclin expression in myocardial tissue, thereby enhancing mitochondrial function, antioxidant capacity, and contractile performance to improve cardiac adaptability.^19^ In an ischemia-reperfusion injury model, exercise preconditioning relies on the Musclin-cyclic guanosine monophosphate (cGMP)/PKGI/CREB/PGC1α signaling axis to activate mitochondrial biogenesis,^71^ and knocking out Musclin completely abolishes the cardioprotective effects of exercise.^70^^,^^71^

**Figure 3:**
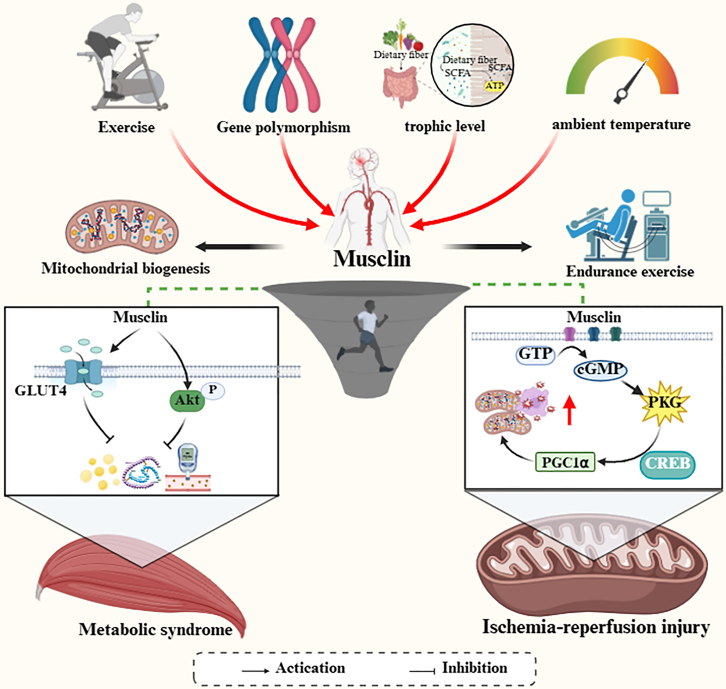
The role of Musclin in exercise adaptation and metabolic regulation

To sum up, Musclin, as a key molecule in the two-way regulation of “metabolism-exercise,” enhances exercise endurance by promoting energy supply in a physiological state, and may cause the desensitization of the signal pathway in a pathological state due to continuous high expression (Figure 3). Future research needs to analyze the following directions: (1) the specific regulatory network of Musclin in different exercise modes (endurance vs. resistance); (2) the mechanism of organ-to-organ communication in its cardioprotection; (3) personalized exercise prescription design based on the dynamic change of Musclin, especially the intervention strategy for patients with metabolic diseases. In addition, the development of functional nutritional supplements or gene editing therapy targeting the Musclin signaling pathway may provide new therapeutic ideas for improving sports performance and preventing MetS.

## Perspectives and conclusion

Musclin, as a specific secretion factor of skeletal muscle, plays a dual role in MetS: on the one hand, as a pathological factor, it aggravates metabolic disorder by interfering with the insulin signaling pathway, inducing ERS, and inhibiting adipose thermogenesis; On the other hand, it may also reflect the adaptive response of the body to metabolic stress as a compensatory sign. It is worth noting that the expression of Musclin is regulated by multiple factors, such as exercise mode, nutritional status, and genetic background, and its functional effect has significant concentration dependence and temporal and spatial specificity. Exercise intervention can improve metabolic health by regulating the dynamic secretion pattern of Musclin: acute exercise induces its pulse elevation and activates beneficial signal pathways, while long-term exercise training can restore its expression to a normal level by improving the overall metabolic state. In addition, Musclin plays an important role in the protection of the heart, and improves heart adaptability by enhancing mitochondrial function and antioxidant capacity. Future research can further explore the specific mechanism of Musclin and how to effectively treat MetS by regulating Musclin level.

Although significant progress has been made in Musclin research, the research of the literature in this field must be approached with caution due to several methodological limitations. First, limited sample sizes and population heterogeneity are major constraints in clinical studies. Most studies have limited sample sizes (*n* < 100), and significant variations in patient age, sex, disease duration, and medication use not only reduce statistical power but may also confound the true association between Musclin and metabolic phenotypes. Second, the lack of standardized detection methods for Musclin is a potential source of contradictory results across studies. Currently used commercial ELISA kits may recognize different Musclin isoforms or exhibit cross-reactivity with osteocrin, making it difficult to compare absolute concentrations between studies. Third, a translational gap exists between animal models and human disease. Current animal studies predominantly use young male animals subjected to acute, severe interventions, which fundamentally differ from the chronic, multifactorial nature of human MetS, thereby limiting the direct clinical applicability of the findings. Finally, exercise intervention protocols lack uniformity. Wide variations in exercise type, intensity, duration, and frequency, along with inadequate control of key confounding factors such as nutrition, complicate efforts to clarify the specific effects of exercise itself on Musclin.

Based on the above limitations and the analysis presented in this review, we propose the following research priorities: (1) Determine the functional switch threshold of Musclin between physiological and pathological conditions and decipher its concentration-dependent regulatory network; (2) Uncover the pathways through which Musclin mediates interactions between skeletal muscle, adipose tissue, liver, and the cardiovascular system, particularly how it coordinates multi-organ dysfunction in MetS; (3) Elucidate the differential effects of endurance and resistance training on Musclin expression and function, and establish precise regulation strategies based on exercise type, intensity, and duration; (4) Explore the feasibility of using Musclin as a biomarker for MetS (e.g., sarcopenia, DN) and develop small-molecule drugs or gene-editing therapies targeting its signaling pathway; (5) Decipher the mechanisms underlying sexual dimorphism in Musclin expression and function, and evaluate the individual impacts of genetic background, age, and drug interventions on its regulation. Future studies should integrate multi-omics technologies, organoid models, and cross-species comparative analyses to systematically clarify the dynamic regulation of Musclin within the metabolism-exercise axis, thereby providing a theoretical foundation and practical guidance for the precise prevention and treatment of metabolic diseases, the optimization of exercise prescriptions, and the development of novel therapeutic targets. In the future, it is necessary to integrate multi-omics technology, organ-like model, and cross-species comparative analysis, systematically clarify the dynamic regulation law of Musclin in the metabolism-exercise axis, and provide theoretical support and practical basis for accurate prevention and treatment of MetS, optimization of exercise prescription, and development of new therapeutic targets.

## Acknowledgments

The work was supported by the 10.13039/100014718National Natural Science Foundation of Young Scholars of China (No.82205147), the 10.13039/100014718National Natural Science Foundation of China (No. 81970261, No. 62472046),the Program of 10.13039/501100003453Natural Science Foundation of Guangdong Province, China (NO.2022A1515010385), Guangdong Provincial Sports 10.13039/100000181Bureau Scientific Research Project (GDSS2024N038), and Research Project on Theory and Practice of 10.13039/100032153Guangdong-Hong Kong-Macao Collaborative Development for the 15th National Games and Special Olympics (2025GBA-524).

## Author contributions

Ruiming Wen: conceptualization and writing- original draft preparation. Yuan Yang: visualization. Haixia Wang: investigation. Yikun Teng: methodology. Bing Zhao: data curation. Hanxiao Zhu: software and validation. Songtao Wang: writing- reviewing, funding acquisition, and visualization.

## Declaration of interests

The authors have declared that no competing interest exists.
